# Pomegranate Germplasm Collections from Elche (Spain) and Bari (Italy): Genetic Resources Characterization for Emerging Mediterranean Challenges

**DOI:** 10.3390/plants14213239

**Published:** 2025-10-22

**Authors:** Ana Lozano-Soria, Agata Gadaleta, Ilaria Marcotuli, Giuseppe Ferrara, Andrea Mazzeo, Julián Bartual, Elena Zuriaga

**Affiliations:** 1 Citriculture and Plant Production Center, Valencian Institute of Agricultural Research (IVIA), CV-315, Km. 10.7, 46113 Valencia, Spain; analozanosoria@gmail.com; 2 Department of Plant, Soil and Food Sciences, University of Bari ‘Aldo Moro’, via Amendola 165/A, 70126 Bari, Italy; agata.gadaleta@uniba.it (A.G.); ilaria.marcotuli@uniba.it (I.M.); andrea.mazzeo@uniba.it (A.M.); 3 Agricultural Experiment Station of Elche (EEA-Elx), Ctra Dolores Km. 1, 03290 Elche, Alicante, Spain

**Keywords:** pomegranate, European, diversity, microsatellite, germplasm, breeding, quality, traits

## Abstract

Pomegranate (*Punica granatum* L.) is a strategic crop for Mediterranean agriculture due to its adaptability to arid environments—an increasingly important trait in the context of climate change—and its rising market demand driven by nutritional and medicinal properties. To support breeding and conservation efforts, this study evaluated the genetic diversity and phenotypic traits of two Mediterranean germplasm collections from Elche (Spain) and Bari (Italy). A total of 184 accessions were analyzed using SSR markers and evaluated for key pomological and phenological traits, including fruit weight, skin and aril color, seed hardness, aril weight, titratable acidity, soluble solids content, and harvest time. Genetic analyses revealed high levels of diversity within and between collections, with clear population structure influenced by geographic origin. Phenotypic evaluation revealed considerable variation in agronomic and quality traits, and several accessions with notably desirable characteristics were identified. For example, Ovadan and Molla Nepes displayed very high soluble solids content (>19 °Bx), Sanrà Nero, Sanrà Rosso, and Tajikistan Dark Red exhibited titratable acidity exceeding 40 g/L citric acid, and De Marco reached aril weights of up to 0.60 g. The integration of molecular and morphological data provided valuable insights into the distinctiveness and breeding potential of the studied genotypes. Misclassifications were identified, as well as phenotypic differences attributable to environmental influences. These findings highlight the importance of characterizing regional germplasm to preserve local adaptations and support development of new cultivars adapted to changing environmental conditions. This work reinforces the role of European pomegranate collections as reservoirs of genetic resources for sustainable cultivation and future breeding programs.

## 1. Introduction

The pomegranate (*Punica granatum* L.) is a perennial fruit species of notable agricultural and medicinal relevance [1]. In recent years, its global cultivation has expanded rapidly, driven by increasing consumer demand for its fruit, which is rich in bioactive compounds and associated with various health benefits [2]. Beyond its nutritional qualities, pomegranate exhibits exceptional adaptability to arid and semi-arid climates, exhibiting a high tolerance to water stress and elevated temperatures [3]. As a result, it stands out as a highly promising crop for the Mediterranean basin and other climate-vulnerable regions increasingly affected by water scarcity and extreme weather events driven by climate change, such as high temperatures.

With a domestication history spanning over 5000 years, pomegranate is among the earliest cultivated fruit crops [4,5]. Over centuries, the domestication and cultivation of pomegranates across a wide geographic range have led to a broad genetic diversity, particularly in traditional cultivars and landraces that have been shaped by local environmental conditions and human selection [6]. Preserving this diversity is essential for future crop resilience, especially considering the urgent need to develop varieties that combine high fruit quality with tolerance to biotic and abiotic stresses. Pomegranate breeding has traditionally focused on selection based on flower, rind, and aril color, fruit size and color, sugar and acid content, stress resistance, yield, and seed hardness [3]. For example, consistent with other initiatives, the pomegranate breeding programs at the Instituto Valenciano de Investigaciones Agrarias (IVIA) and the Department of Soil, Plant and Food Sciences (DiSSPA) at the University of Bari focus on developing new cultivars that extend the harvesting season, with red to violet fruits of subacid flavor, soft seeds, good fruit size, and high yield, while ensuring tolerance to diseases such as Alternaria and to physiological disorders like cracking, as well as good storability and superior commercial quality. In this context, germplasm repositories play a critical role in safeguarding this genetic reservoir. However, the utility of conserved materials depends on their proper characterization—both genetically and phenotypically—to uncover valuable traits and guide their use in breeding or direct cultivation.

Several ex situ initiatives have been established to conserve pomegranate diversity [7], with prominent examples including the Garrygala Research Station in Turkmenistan and the Vavilov Research Institute in Russia. In Europe, key collections are housed at the Agricultural Experiment Station of Elche (EEA-Elx, Alicante, Spain), a public institution under the Regional Government of Valencia, and at the University of Bari ‘Aldo Moro’ (Bari, Italy). Both collections include wild relatives, landraces, traditional varieties and cultivars, advanced breeder selections, and hybrids, offering valuable resources for developing new cultivars adapted to Mediterranean (or similar) environments. However, effectively leveraging these resources requires systematic evaluation, a task made particularly challenging by the often-limited morphological differences among closely related accessions and/or varieties. Detecting these subtle differences (if any) requires increasingly detailed phenotyping or targeted assessments of specific traits. In the omics era, phenotyping has become a major bottleneck, not only for breeding but also for genetic analyses in general.

Pomegranate genetic diversity has been extensively studied in countries where the species plays a prominent historical, cultural and economic role, such as Turkey [8], Iran [9,10], India [11], Pakistan [12], Spain [13], and Italy [14]. Despite this broad interest, a comparative analysis of the germplasm conserved in the Bari and Elche repositories had not been undertaken until the present study. Such studies are crucial for developing core collections that capture genetic breadth while minimizing redundancy, preserving rare genotypes at risk due to environmental pressures or genetic erosion, and selecting genetically distinct individuals for breeding to introduce novel allele combinations.

In this context, the present study provides the first integrated genetic and phenotypic analysis of the Bari and Elche collections. Using microsatellite markers (SSRs) alongside key pomological trait assessments, we examine the genetic structure, diversity, and agronomic potential of these accessions. Our findings aim to identify unique or high-performing genotypes for breeding and cultivation under Mediterranean pedoclimatic conditions, contributing to improved germplasm management and the long-term sustainability of pomegranate production in climate-challenged regions.

## 2. Results

### 2.1. Pomological Diversity Maintained in Both Germplasm Collections

As explained, the Bari and Elche collections represent a global effort to preserve and study the genetic diversity of pomegranate varieties, with the aim of improving and enhancing the species in the future. In this study, we analyzed eight key pomological traits from 90 accessions in the Bari collection and 94 from Elche, together with their classification as ornamental and their putative origin (Table S1). The dataset also includes 22 accessions shared between both collections. In addition, accessions were classified as either cultivars or landraces, a distinction intended primarily to emphasize the extent of local versus broader use of the varieties rather than to provide a strict taxonomic categorization. However, the differentiation between landraces and cultivars remains a subject of ongoing debate, even within horticultural species [15], and this ambiguity is often greater in vegetatively propagated fruit crops such as pomegranate.

**Figure 1:**
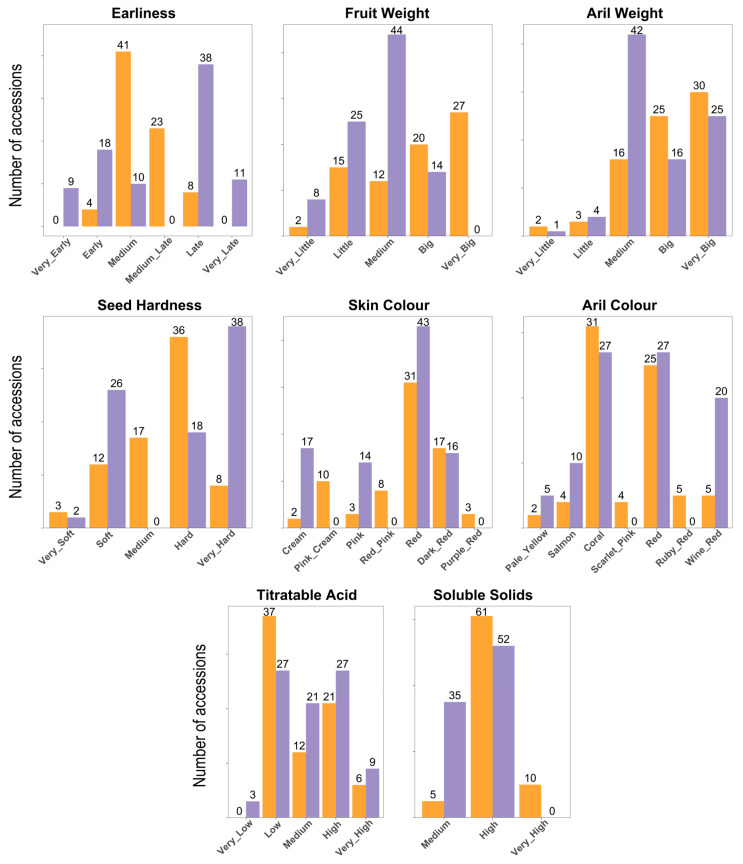
Phenotypic variation among 184 pomegranate accessions from the Bari (mustard yellow) and Elche (lavender) collections. The number of accessions for each type is indicated.

**Figure 2:**
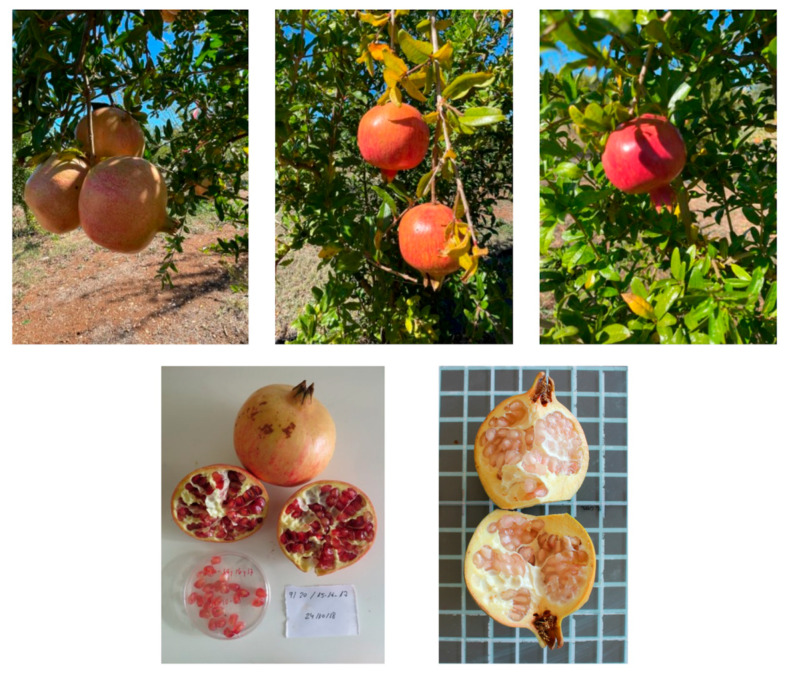
Representative depiction of the morphological variation documented in both collections. The upper row shows, from left to right, fruits from Specchia, Casamassima, and Dolce Corallo. The lower row shows open fruits of Specchia and White Flower, highlighting the color of the arils in detail.

As the characterization followed UPOV descriptors in both collections, and taking into account the presence of the common samples, a standardization process to ensure consistency in the merged data (Table S2). It should be noted, however, that in some cases differences were observed, likely due to environmental conditions affecting the duplicated samples in the two collections. For example, ‘Acco’ and ‘Kaim Anor’ exhibited a darker fruit peel color in Bari than in Elche. The distribution of key characteristics of the analyzed accessions is presented in Figure 1. In general, Bari accessions exhibit a wide variation in fruit weight, ranging from very small to very large, whereas the accessions from Elche typically produce fruits of smaller to medium size. Regarding skin color, both collections feature red and dark red fruits; however, the Bari collection also includes lighter colors such as pink, cream, and yellow-pink, which are less common in the Elche collection (Figure 2). The Elche collection exhibits a broader harvest window, not only due to the presence of very early-ripening accessions—maturing up to 10 days earlier than in Bari (i.e., before 20 August)—but also due to a higher number of late and very late ones. The Elche collection includes 9 very early and 18 early accessions, whereas the Bari collection contains 4 early accessions. Furthermore, Elche holds a substantially greater number of late and very late accessions (38 and 11, respectively), compared to just 8 late accessions in Bari. Concerning seed hardness, both collections include materials with soft or very soft seeds, which could be promising for breeding purposes. Aril weight in both collections shows considerable variation, ranging from small arils, such as 0.15 g in ‘Sanrà Nero’, to very large arils, such as 0.60 g in ‘De Marco’. Regarding aril color, the Elche collection contains more accessions with extreme color variations, such as clear or dark shades. Regarding titratable acidity, considerable variability was observed in both collections. The Bari collection predominantly includes accessions with low acidity levels, although some cultivars exceed 40 g/L of citric acid, such as ‘Sanrà Nero’ (46 g/L), ‘Sanrà Rosso’ (48 g/L), and ‘Tajikistan Dark Red’ (45 g/L). Finally, several accessions from the Bari collection exhibited very high °Bx values, including the Italian landraces ‘Antico Macello’ and ‘Battista’, among others.

Correlation analysis reveals several expected patterns among the measured traits (Figure S1). Fruit Weight and Aril Weight are moderately positively correlated (0.38), indicating that larger fruits tend to have heavier arils. Skin Color and Aril Color are strongly correlated (0.60), reflecting a consistent relationship between external and internal pigmentation, and Skin Color also shows a moderate positive correlation with TA content (0.27). The Shannon–Wiener diversity index is a commonly used measure to quantify the variability or diversity within a set of traits, considering both the number of categories and their relative abundance. In our study, this index revealed notable differences in phenotypic variation across collections and regions (Table S3). Between the Bari and Elche collections, Skin Color was more diverse in Bari (1.58 vs. 1.26), while Harvest Time showed higher diversity in Elche (1.45 vs. 1.09). Aril Color was similarly diverse in both collections (Bari 1.50, Elche 1.47), and Soluble Solids and Type exhibited moderate diversity (0.62–0.67 and 0.60–0.65, respectively). Across geographic regions, diversity patterns varied by trait: Fruit Weight was highest among accessions from North America (1.53) and the Mediterranean Basin (1.44) but absent in materials from Central and South Asia (0). Skin Color exhibited peak diversity in the Mediterranean (1.66) and North America (1.60). Harvest Time and Seed Hardness were most variable in Central Asia and the Mediterranean, while Aril Weight and Aril Color were highly diverse in North America and Central Asia but low in East Asia. TA content reached its maximum diversity in North American accessions (1.43). Overall, these results highlight substantial regional differences, with North America and the Mediterranean consistently exhibiting higher phenotypic variation, and underscore the complementary value of the Bari and Elche collections in capturing pomegranate diversity.

Both collections include ornamental accessions (4 in Bari and 9 in Elche), with some shared between the two collections, such as ‘Dotch Legrelley’, ‘Haku Botan’ (that means ’White Peony’ in Japanese), ‘Ki-Zakuro’ (‘Yellow pomegranate’ in Japanese), and ‘White Flower’. Regarding ‘Nochi Shibori’ and ‘Toryu-Shibori’, first one can be translated as ‘Late Shibori’ or ‘Late Dyeing’, while ‘Toryu-Shibori’ could be interpreted as ‘Eastern Dragon Shibori’. Both names include the term ‘Shibori’, which in Japanese refers to a traditional fabric-dyeing technique that creates intricate patterns, suggesting that these varieties might have mottled or variegated flower colors.

### 2.2. Genetic Diversity, PIC and Cultivar Identification

In this study, the germplasm from the Bari collection was genotyped using 16 SSR markers (Table S4), selected from Soriano et al. [16]. For each PCR reaction, a set of reference accessions from the Elche collection was included to standardize the results and enable their integration with those obtained in a previous study [13]. The combined analysis of both collections confirmed that all 16 SSR markers were polymorphic in both datasets (Table S5). The 16 selected SSR markers enabled the identification of 92 distinct alleles and 139 unique haplotypes among the 184 studied accessions. The number of detected alleles for each marker varied from 3 (PGCT016, PGCT032 and PGCT057) to 13 (PGCT110A), with an average of 5.75. PIC values ranged from 0.180 (PGCT022) to 0.655 (PGCT111), with a mean value of 0.51 (Table S4). Twenty-six rare alleles, considered as polymorphic alleles having <1% frequency, have been identified (7 with PGCT110A, 3 with PGCT015 and PGCT022, 2 with PGCT088A, PGCT089, PGCT093B and PGCT111, and 1 in the case of PGCT066, PGCT028, PGCT083, PGCT087 and PGCT091). Observed heterozygosity ranged between 0.185 (PGCT022) to 0.614 (PGCT111), while nonbiased expected heterozygosity [17] ranged between 0.180 (PGCT022) to 0.656 (PGCT111). Hnb estimates the probability that two randomly chosen alleles are different, reflecting genetic diversity at a locus while correcting for sample size bias.

Since both collections share 22 accessions with matching names, the marker pattern was analyzed to verify their consistency, revealing some discrepancies (Table 1). Six accessions had identical profiles, while 11 differed just by one or two alleles. However, five accessions showed distinct profiles in four SSRs (‘Kaj Acik Anor’), nine SSRs (‘Azadi’ and ‘Dotch Legrelley’), or ten SSRs (‘Molla Nepes’ and ‘Cranberry’). This suggests a high likelihood of misclassification within one of the germplasm collections, warranting further review.

Within the full set of accessions, 23 small groups were identified in which accessions shared identical SSR marker profiles (Table 2). Sixteen of these groups consisted of just two accessions, including those shared between both germplasm collections—except for ‘Akko’/‘Acco’, which clustered with ‘Emek’. Three additional groups contained four accessions each, one group included six, and two groups comprised eight accessions. Some of these redundancies had already been described in a previous study of the Elche collection [13]. Notably, groups G20, G21, and G22 included 4, 6, and 8 Italian landraces, respectively. Group G23, the largest cluster, contained a mixture of landraces and varieties from different origins. To minimize bias in downstream analyses, only one representative accession from each group was retained for subsequent analyses.

### 2.3. Population Structure and Genetic Relationships

**Figure 3:**
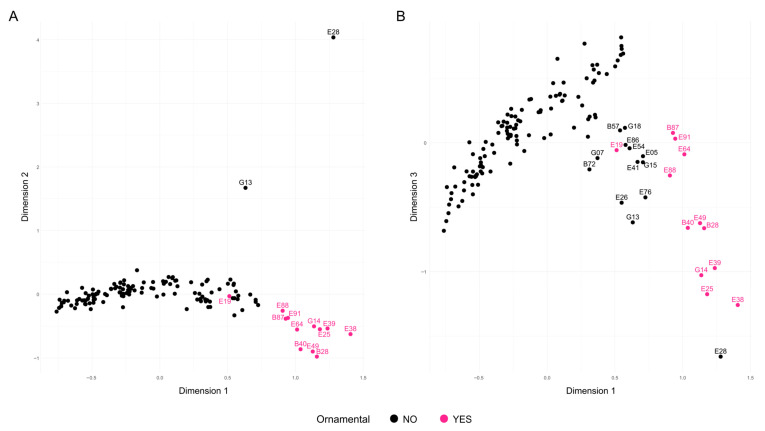
Factorial Correspondence Analysis (FCA) of the 140 unique genotypes analyzed using 16 SSR markers. Accessions are shown in black, except for the ornamental ones, which are highlighted in pink. (**A**) Projection onto Dimensions 1 and 2; (**B**) Projection onto Dimensions 1 and 3.

Three different approaches were used to investigate the relationships between accessions and population structure. First, several Factorial Correspondence Analyses (FCAs) were performed to explore the relationships among the samples. For clarity, since only one representative was kept for each group of samples with identical genotypes (redundant samples), the group name, as it appears in Table 2, was used to represent these samples. The initial FCA, which included all 140 accessions after removing potential redundancies, explained 23.64% of the total variability across the first three dimensions (Figure 3). The three accessions from India—B08 and B13 (both grouped as G13), and E28—appeared clearly separated from the rest along the second dimension, with ‘Elx-13’ (E28) showing even greater divergence along the third dimension. Additionally, accessions classified as ornamental were grouped and distinct from the others, except for the North American cultivar ‘Chico’ (E19), which appeared in a central position (Figure 3A). A group of eight ornamental accessions appeared on the right side of the first dimension and were further separated along the third dimension (Figure 3B). These included ‘Ki-Zacuro’ from Bari (B40) and Elche (E49), ‘Dotch-Legrelley’ from Bari (B28) and Elche (E25), ‘Haku Taka’ (E39), ‘Haku Batan’ (E38), and G14, which comprises ‘Haku Botan’ (B36) and ‘How sweet it is’ (E40). All these accessions are of Japanese origin, except for the two ‘Dotch-Legrelley’ samples, which are reported to originate from Turkmenistan according to passport data, and ‘How sweet it is’, whose origin remains unknown.

**Figure 4:**
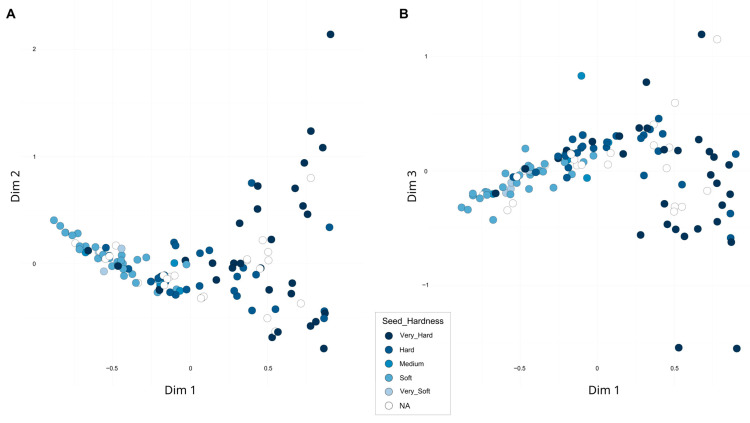
Second Factorial Correspondence Analysis (FCA) based on 16 SSR markers. Accessions are colored according to seed hardness. (**A**) Projection onto Dimensions 1 and 2; (**B**) Projection onto Dimensions 1 and 3.

A second FCA, excluding the three samples from India as well as the distant ornamental accessions, accounted for 22.58% of the total variability across the first three dimensions (Figure 4). When assessing whether the distribution of accessions was related to previously analyzed pomological traits or their geographic origin, a clear association with seed hardness was observed. Along the first dimension, accessions with harder seeds tended to cluster on the right, while those with softer seeds were located on the left. Overall, hard-seeded accessions exhibited greater dispersion, whereas soft-seeded types appeared more tightly clustered. A detailed plot including the accession codes is presented in Figure S3. Notably, the accession ‘Shainakskii’ (E76) was distinctly separated along the second dimension and was close along the third dimension to ‘Kaj Acik Anor’ (E45), an accession from the former Soviet Union. In contrast, at the opposite end of the third dimension, ‘Hyrdanar × Goulosha’ (E41) was positioned separately, closely associated with group G15, composed of ‘Muagkosemyannyi rozovyi’ (B51) and ‘Hyrdanar × Kirmizy–Akbuh’ (E42). Slightly more distant from this cluster, though still within the same region of the third dimension, were the accessions ‘Turi’ (B83) and ‘Sanra Nero’ (B72).

**Figure 5:**
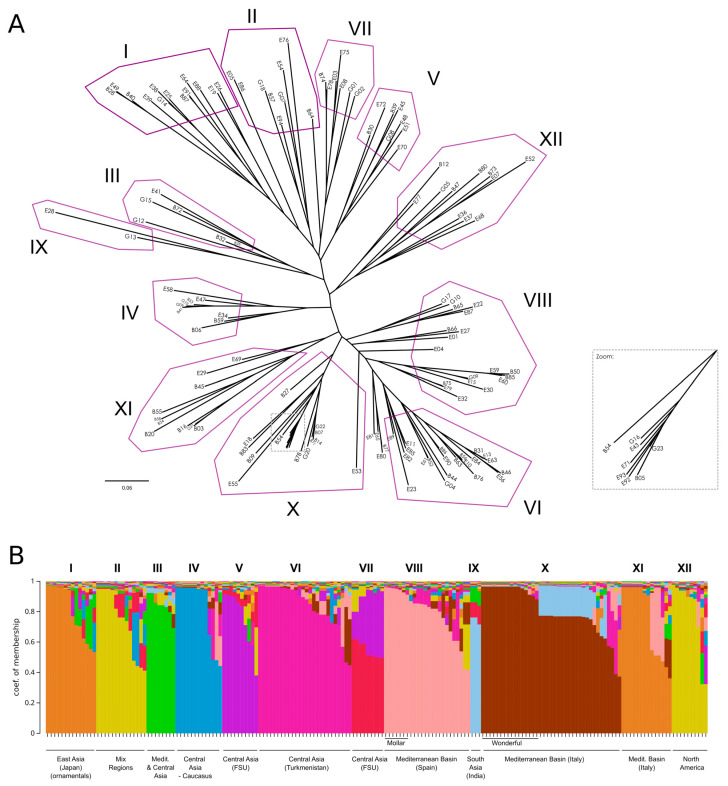
(**A**) Neighbor-Joining tree using Cavalli-Sforza and Edwards’ distance [18]. A zoomed-in section is shown for clarity. (**B**) Bayesian-based population assignment allowing admixture carried out using the Structure software (K = 12 based on the maximum likelihood of K).

As a complementary approach to investigate relationships among the accessions, we constructed a Neighbor-Joining tree using Cavalli-Sforza and Edwards’ distance [18] (Figure 5A). Overall bootstrap support values were low, with only a few peripheral nodes exceeding 50; for a more detailed view of bootstrap values, please refer to the phylogenetic tree in Supplementary File S1. Next, we performed a Bayesian population-assignment analysis allowing admixture with STRUCTURE 2.3.4 software [19]. Various methods exist to determine the optimal number of clusters (K) [19,20]. In our case, the ΔK method indicated K = 2 as the most likely, whereas the highest log-likelihood corresponded to K = 12 (Figure S4). By examining accession assignments at K = 12 (Figure 5B, Table S6), we found that this clustering broadly recapitulates the phylogenetic tree, with accessions aligning to their Structure clusters despite relatively weak inter-cluster separation. Specifically, clusters I, II, V, and VII form a tight group, while clusters VI, VIII, X, and XI form another block at the opposite end of the tree. The remaining clusters lie between these two main groups. Figure S4 provides a detailed overview listing the names of the samples in each cluster. The clustering patterns show a broad, though not strict, association with the geographic origin of the accessions. However, the absence of well-defined geographic structuring across clusters indicates a high degree of historical gene flow and germplasm exchange in this species since the Phoenicians or even earlier. Despite this overall admixture, certain groups—such as the ‘Mollar’ and ‘Wonderful’-type accessions—exhibit clearer genetic coherence, potentially reflecting more localized selection or domestication events.

Remarkably, ornamental accessions formed a well-supported Cluster I, confirming their unique genetic identity. This cluster included matching entries from both collections, such as ‘Dotch Legrelley’ (E25 and B28), ‘Ki-Zakuro’ (B40 and E49), and ‘Haku Botan’ (B36 and E38), which also grouped together in the FCA. Interestingly, ‘Elf’ (E26) from the Elche collection—initially not classified as ornamental—was placed within this cluster, close to ‘Chico’ (E19), with both at a basal position in the ornamental clade. Cluster II is genetically diverse, comprising accessions from multiple regions (Asia, Eastern Europe, and North America), and is characterized by the predominance of large and firm-textured fruits. Cluster III shows moderate genetic diversity, with accessions mainly originating from Central Asia and the Mediterranean Basin. Phenotypically, it is characterized by predominantly medium to large fruits, with hard to very hard textures, and a wide range of skin colors, where red and coral tones are most common. Cluster IV consisted primarily of Central Asian accessions, with a few from Transcaucasia and North America, a mix of traditional and improved germplasm. These accessions typically bore medium-to-very-large fruits with firm to very firm textures. Cluster V was dominated by Central Asian, former Soviet Union accessions. Phenotypically, fruit sizes and skin colors were variable, although almost uniformly very hard in texture. Cluster VI comprised mostly Central Asian accessions—especially from Turkmenistan—with a few from North America. They produced large to very large fruits with soft to very soft textures. Cluster VII includes accessions primarily from Eastern Europe and Central Asia, mostly from the former Soviet Union. The accessions bear medium to very large fruits, typically with hard or very hard textures. This group is mainly composed of cultivars, indicating a history of selection and breeding in continental temperate regions. Cluster VIII comprised Mediterranean (mainly Spain), Middle Eastern (Israel), and Central Asian accessions, both traditional landraces and modern cultivars. Fruits were generally soft to very soft, red, coral, or pink, and the group included early-maturing types adapted to warm climates and markets favoring sweet, easy-to-eat fruits—most notably Mollar-type varieties from the Elche collection. Although internal bootstrap support was weak, the tight grouping of Mollar-type accessions suggests a shared genetic background shaped by regional selection. Indian cultivars—‘Bhagawa’ (B13), ‘Arakta’ (B08), and ‘Elx-13’ (E28)—formed a distinct branch in Cluster IX, underscoring their genetic divergence and corroborating the FCA results. Cluster X brought together genetically diverse accessions from Italy and Turkmenistan, encompassing both landraces and modern cultivars, including varieties related to the esteemed ‘Wonderful’ type. This group includes several genotypes labeled ‘Wonderful’, a widely cultivated cultivar in North America and Europe, showing consistent genetic identity despite minor phenotypic variation. Cluster XI comprised primarily Italian landraces (especially from Bari province) and the Iranian cultivar ‘Mahali Dezful’ (E55). Italian accessions produced medium to very large fruits with red to pinkish skins and arils, maturing medium-late to late with firm to very firm textures. The Iranian accession yielded smaller fruits yet very firm and late-maturing, highlighting strong local adaptation and diverse traits valued in regional markets. Finally, Cluster XII included modern North American cultivars (mostly U.S. origin), one Italian, one Iranian, and several with uncertain provenance. These accessions varied in size and color but typically matured late and exhibited firm to very firm textures.

Lastly, four accessions from different Structure clusters converge at a basal position relative to Cluster VI and adjacent to Cluster VIII: the two ‘Sogdiana’ entries from Bari (B77) and Elche (E80)—assigned to Clusters VI and X, respectively—are grouped with ‘Orange’ (E65) and ‘Sumbar’ (E81). While ‘Sogdiana’ and ‘Sumbar’ both originate from Turkmenistan, ‘Orange’ has an unknown provenance. All four accessions, sourced from the USDA germplasm repository, produce exceptionally large, visually striking fruit—‘Sogdiana’ with glossy red rinds, ‘Orange’ with vibrant orange-red skins, and ‘Sumbar’ with pale-red exteriors—and feature tender, soft arils that are easy to eat. Their well-balanced sweet–tart flavor makes them highly desirable for fresh consumption, and they are also valued as ornamental varieties.

## 3. Discussion

The pomegranate holds deep historical and cultural significance [6] and has attracted growing scientific interest due to its rich phytochemical profile, antioxidant properties, and documented health benefits [2]. In this context, our study offers a comprehensive genetic and phenotypic characterization of two key germplasm collections from Elche (Spain) and Bari (Italy), representative of strategic Mediterranean regions that are increasingly vulnerable to the effects of climate change. Both countries possess a long-standing tradition of pomegranate presence and cultivation of more than two thousand years [21], and rank among the world’s leading producers. This agricultural heritage has fostered the development of locally adapted varieties, valuable for breeding programs targeting improved resilience and fruit quality under changing environmental conditions.

The joint characterization of the Bari and Elche collections reveals substantial, partly complementary genetic diversity, highlighting their combined importance for both conservation and breeding purposes. The integration of molecular and phenotypic data proves particularly valuable for resolving varietal identities, detecting redundancies, and identifying elite accessions with promising agronomic traits, especially when phenotypic variation alone is insufficient to discriminate among certain varieties. Moreover, evaluating material grown under diverse environmental and management conditions is particularly challenging. Indeed, our results emphasize the influence of pedoclimatic conditions on fruit ripening and quality, since even among some accessions shared between the two collections, some notable phenotypic differences are observed, likely reflecting environmental effects. For instance, temperature has been shown to influence titratable acidity, soluble solids, and pH, as observed in Iranian cultivars grown under contrasting cool and warm climates [22]. Harvest timing likewise affects both physical and chemical traits: late harvests typically lead to increased fruit weight, soluble solids, pH, and anthocyanin content, while reducing total phenols, as reported for ‘Wonderful’ across different orchards [23]. Moreover, orchard management practices—such as mulching, pruning, and training systems—can further shape yield and fruit quality [24]. Taken together, these factors highlight the critical value of molecular markers.

Notably, discrepancies among accessions sharing the same name—some showing clear genetic differences across multiple SSR loci—highlight persistent challenges in germplasm curation, a problem also documented in other long-lived crops such as grapevine [25] and olive [26]. Accurate molecular fingerprinting is therefore essential to minimize mislabeling and redundancy, which can otherwise limit the efficient use of genetic resources. Although both collections had been previously analyzed independently [13,27], our joint analysis confirms that they harbor extensive genetic variation. A total of 139 unique genotypes and 92 alleles were identified across 16 SSR markers, reinforcing earlier findings that highlight the heterogeneity of pomegranate germplasm [8,15,28,29]. The presence of rare alleles and elevated heterozygosity, particularly in accessions from Mediterranean and Central Asian origins, reflects the long history of cultivation and human-mediated selection in these regions. Our findings are consistent with those reported by Polat et al. [30], who detected 177 alleles in 127 Turkish genotypes using 34 SSR markers, with an average PIC of 0.48—a value closely matching the mean PIC of 0.50 observed in this study. Such consistency, despite differences in marker sets and genetic backgrounds, suggests that Mediterranean pomegranate germplasm maintains a relatively stable and well-preserved genetic base. These results also affirm the robustness of SSR markers for diversity assessment in this fruit crop.

Phenotypic evaluation revealed broad variability in traits of agronomic and commercial relevance, including aril weight, seed hardness, skin pigmentation, and juice sweetness. Other traits, such as bud and flower characteristics—also important from a commercial perspective [31]—may further differentiate pomegranate varieties of diverse origin. Several accessions combine favorable traits—such as soft seeds and high soluble solids content—making them promising candidates for breeding under Mediterranean conditions or similar environments, consistent with previous reports highlighting the importance of these traits for consumer acceptance and market potential [32,33].

Furthermore, the partial correlation observed between genetic structure and phenotypic traits—such as seed hardness patterns aligning with FCA clustering—suggests that marker-assisted selection strategies could be implemented effectively as more detailed genomic resources become available. Notably, hard-seeded accessions demonstrate greater phenotypic diversity, while soft-seeded ones form more cohesive clusters, likely reflecting domestication and selective breeding influences and implying a genetic relatedness among soft-seeded types. Consequently, these accessions represent valuable material for further genomic investigations, such as those conducted by Luo et al. [34], to elucidate the genetic basis underlying these phenotypic distinctions.

Genetic structure analyses revealed clearly defined groups, largely corresponding to cultivar type or geographic origin. Several of these groups exhibited strong consistency across Bayesian clustering, factorial correspondence analysis, and phylogenetic reconstruction, reflecting well-established patterns of domestication, clonal propagation, and regional adaptation. For instance, ornamental accessions, particularly those of East Asian origin, formed a distinct and genetically homogeneous clade, as previously reported [13]. Likewise, Mollar-type cultivars—a population variety of high economic relevance in Spain—were clearly differentiated, as were ‘Wonderful’ accessions, whose pronounced genetic uniformity is consistent with their widespread clonal propagation in global commercial orchards [1,35].

Looking ahead, the integration of next-generation sequencing, together with QTL mapping and functional genomics, will provide deeper insights into the heritability of key traits, genotype-by-environment interactions, and phenotypic plasticity. Strengthening international collaboration will be crucial to harmonize phenotyping protocols, improve germplasm databases curation, and facilitate data sharing across collections [36]. These coordinated efforts are particularly important for underutilized crops like pomegranate, where joint initiatives can significantly accelerate the development of resilient, high-quality cultivars adapted to Mediterranean and semi-arid conditions [37]. In this sense, our work represents a further step toward fostering international collaboration and advancing the collective knowledge on this underexploited species.

## 4. Conclusions

This study underscores the rich genetic and phenotypic diversity preserved in two major European pomegranate collections from Elche (Spain) and Bari (Italy). Pomegranate accessions showed a great variability of almost all fruits parameters, including color, soluble solids content and acidity, very important traits for consumer’s acceptance. Through the combined use of microsatellite markers and pomological trait analysis, we identified unique genotypes, redundancies, and accession misclassifications, enhancing the basis for more efficient germplasm management. Our results highlight the importance of integrating molecular and phenotypic data to support breeding efforts focused on resilience, fruit quality, and climate adaptation. This work also emphasizes the need for ongoing international collaboration and the adoption of advanced genomic tools to broaden diversity studies. The characterized resources offer valuable potential for the sustainable improvement of pomegranate cultivation across Mediterranean and similar environments.

## 5. Materials and Methods

### 5.1. Plant Materials and Environmental–Agronomic Background of Germplasm Banks

In this study, 184 pomegranate accessions from diverse origins were analyzed (Table S1), including 22 accessions shared between both collections. In some cases, accession names show slight variations, likely due to transcription errors during data entry in the respective repositories. Both institutions have imported plant material from other germplasm banks and have also conducted local collection expeditions and surveys.

Of these, 94 accessions belong to the collection maintained since 2010 at the Agricultural Experiment Station of Elche/Valencian Institute of Agricultural Research (EEA-Elx/IVIA), under the jurisdiction of the Valencian regional government, located in Elche, Alicante province, southeastern Spain. Elx and Elche are the Valencian and Spanish names, respectively, of the same city. For the sake of clarity and consistency with other academic works, both denominations are used interchangeably and considered equivalent. This region has a semi-arid Mediterranean climate, characterized by long, hot, dry summers with temperatures often exceeding 30 °C in July and August, and mild winters that rarely drop below 6 °C. Annual precipitation is low, around 300 mm, mostly concentrated in autumn (October) and occasionally in spring (April). Pomegranate trees in the orchard were planted at 4.5 × 3.5 m spacing on calcaric fluvisol soil (sandy loam; pH 8.4; effective depth >120 cm). Irrigation water had an average electrical conductivity (EC) of 1.19 dS m^−1^ at 25 °C, and annual fertilization rates were 90, 45, and 120 kg ha^−1^ of N, P_2_O_5_, and K_2_O, respectively. Cultural practices followed local standards.

The remaining 90 accessions are part of the fruit collection conserved at the pomegranate repository of the Fruit Tree Unit within the Department of Soil, Plant, and Food Science (DiSSPA) at the University of Bari ‘Aldo Moro’, Italy. These trees have been cultivated since 2013 in the countryside of Valenzano (Bari, Italy) at the ‘P. Martucci’ experimental and educational center. This area has a typical Mediterranean climate, with an average annual temperature of 15–16 °C, summers averaging 25–30 °C, and winters 6–10 °C, with an annual rainfall of about 550 mm. Further details on the climatic characteristics of the site can be found in previous studies [32,37]. The soil of the pomegranate repository is characterized by a pH of 7.7, EC 0.22 dS m^−1^, organic carbon content of 11.4 g kg^−1^, and nitrogen content of 1.3 g kg^−1^ [38].

Regarding the origin of the samples, the Elche collection analyzed in this study comprises 72 accessions imported from the National Clonal Germplasm Repository for Tree Fruit, Nut Crops, and Grapes in Davis (CA, USA), 1 from the Newe Ya’ar Research Center (Ramat Yishay, Israel), and 13 obtained through collection expeditions and crosses carried out by EEA-Elx researchers. Meanwhile, the Bari collection analyzed here includes 17 accessions from the Davis germplasm, with the remainder originating from their own expeditions and surveys. The origin of the accessions is quite diverse, from regions such as Central Asia (mainly Turkmenistan), the Mediterranean, East Asia, North America, South Asia, the Caucasus, and the Middle East. The Bari collection contains 41 landraces (local varieties that have evolved in their native regions since centuries) and 20 cultivars (selected varieties cultivated for specific traits), while the Elche collection includes 6 landraces and 79 cultivars, making a total of 47 landraces and 99 cultivars across both collections.

For DNA isolation, two leaf disks were collected from each accession, frozen in liquid N2 and stored at −80 °C until use.

### 5.2. Morphological and Pomological Characterization

Eight key traits relevant to pomegranate breeding were evaluated and standardized across both germplasm collections (Table S1). In both collections, data was collected from 2022 to 2024. The 22 shared accessions were used as a reference to standardize phenotypic traits between the two environments. Fruit and aril weights were measured in grams using a precision digital scale, seed texture was evaluated through chewing resistance by trained evaluators and categorized. Juice quality was determined in 3 replicates of 5 fruit each per treatment. Soluble solid content of the juice was measured using a digital refractometer (model PR1; Atago Co., Ltd., Tokyo, Japan) and values were expressed as °Bx. The titratable acidity (TA) of the fruit juice was determined by titrating 1 mL of juice sample with 0.1 mol L^−1^ sodium hydroxide to an end point of pH 8.1 and expressed as g L^−1^ of citric acid. Accessions were classified into five ripening categories: very early, early, medium, late, and very late, with the dates adjusted between the two collections to account for environmental differences. Table S2 summarizes the categories defined for each phenotypic trait, with a reference accession provided for each case and germplasm collection. The traditional use of some varieties as ornamentals has also been indicated.

### 5.3. DNA Isolation and Microsatellite Analysis

DNA extraction was carried out using the method described by Doyle and Doyle [39]. Quantification was performed with a NanoDrop ND-1000 spectrophotometer (Thermo Fisher Scientific, Wilmington, DE, USA), and DNA integrity was assessed on a 1% agarose gel. Sixteen SSR markers (Table 1) were selected based on their informative content, as reported by Soriano et al. [16]. SSR amplifications were conducted in a final volume of 20 µL, containing 1× DreamTaq buffer, 0.2 mM of each dNTP, 20 ng of genomic DNA, and 1 U of DreamTaq DNA polymerase (Thermo Fisher Scientific, Waltham, MA, USA), using a VWR UNO96 Thermocycler (VWR, Radnor, PA, USA). Each reaction included three primers: a specific forward primer for each microsatellite with an M13(−21) tail at its 5′ end (0.05 µM), a sequence-specific reverse primer (0.25 µM), and a universal fluorescent-labeled M13(−21) primer (0.2 µM) [40]. PCR cycling conditions were as follows: initial denaturation at 94 °C for 2 min, followed by 35 cycles of 94 °C for 30 s, annealing at an optimized temperature [16] for 60 s, and extension at 72 °C for 90 s, concluding with a final extension at 72 °C for 10 min. Allele lengths were determined using an ABI Prism 3130 Genetic Analyzer (Thermo Fisher Scientific, Waltham, MA, USA), with fragment analysis performed using GeneMapper software, version 4.0 (Applied Biosystems, Waltham, MA, USA). The genome positions of the SSR markers were determined using the National Center for Biotechnology Information’s (NCBI) BLASTn tool by comparing the primer sequences against the ASM765513v2 genome assembly, which served as the reference.

### 5.4. Data Analysis

Histograms of morphological and pomological traits were generated in R [41]. Bar plots were created using ggplot2 [42] and patchwork [43] packages. Shannon–Wiener diversity indices were calculated per trait and per genebank using the diversity function from the vegan package v. 2.7.1 [44]. Spearman correlations among phenotypic traits were computed with dplyr [45] and reshape2 [46], and heatmaps were used to visualize pairwise relationships created using ggplot2.

Regarding microsatellite markers, the number of alleles, their size range, and polymorphism information content (PIC) were calculated for each one. PIC was determined based on allele frequencies across all analyzed cultivars using the formula: PIC*i* = 1 − Σp*ij*^2^, where p*ij* represents the frequency of the *j*th allele at the *i*th marker locus, summing over all alleles. Observed (Ho) and expected (He) heterozygosity [17] were calculated using the Genetix software v. 4.05.2 [47]. To assess the relationships among accessions, several factorial correspondence analyses (FCA) were performed also using Genetix. Genetic distances between accession pairs were calculated based on Cavalli-Sforza and Edwards’ distance [18] and used to construct an unrooted neighbor-joining (NJ) phylogenetic tree, using the R package Poppr v. 2.9.8 [48]. Node stability was evaluated through 1000 bootstrap replicates. Tree visualization was performed with HyperTree software v. 1.2.2 [49]. Accessions were further classified into genetic clusters using the Bayesian model-based clustering approach implemented in STRUCTURE 2.3.4 [19], under the admixture model with unlinked loci, correlated allele frequencies across groups, and no prior population information. Twenty independent runs were performed for each predefined number of populations (K) ranging from 1 to 13, using a burn-in period of 100,000 iterations followed by a post-burn-in simulation of 1,500,000 iterations per run. The optimal K-value was determined using Structure Harvester [50], based on the log probability of the data [LnP(D)] and the ΔK method, which considers the rate of change in [LnP(D)] between successive K-values. For the selected K-value, membership coefficient matrices from 20 replicates were merged using CLUMPP v. 1.1.2 [51] to generate a consensus Q matrix. STRUCTURE PLOT software v. 2.0 [52] was used to visualize STRUCTURE bar plots.

## Figures and Tables

**Table 1:** Accessions shared between both germplasm collections that exhibit differences in any of the analyzed SSR markers. The accession name, sample number corresponding to each collection, and the number of markers showing different profiles are indicated. NA = Not Available.

Name	Acc Number Bari	Acc Number Elche	No. Different SSR
Acco	B04	E02	0 (4 NA)
Cana	B15	E16	0
Gissarskii Rozovyi	B35	E35	0
Kaim Anor	B38	E44	0
Parfyanka	B63	E67	0
Surh-anor	B79	E83	0
Ki-Zakuro	B40	E49	1
Myatadzhy	B52	E61	1
Palermo	B60	E66	1
Purple Heart	B67	E71	1
Sirenevyi	B75	E79	1
Sogdiana	B77	E80	1
White Flower	B87	E91	1
Haku Botan	B36	E38	2
Medovyi Vahsha	B46	E56	2 (1 NA)
Shirin Zigar	B74	E78	2
Vkusnyi	B86	E90	2
Kaj Acik Anor	B39	E45	4
Azadi	B09	E11	9
Dotch Legrelley	B28	E25	9
Cranberry	B22	E21	10
Molla Nepes	B49	E58	10

**Table 2:** Groups of accessions sharing identical genetic profiles based on the 16 SSR markers used in this study.

Group N.	Acc.	Name	N. accs	Observations
G01	B38; E44	Kaim anor (Bari), Kaim anor (Elche)	2	Shared accessions
G02	B79; E83	Surh anor (Bari), Surh-anor (Elche)	2	Shared accessions
G03	B63; E67	Parfianka (Bari), Parfyanka (Elche)	2	Shared accessions
G04	B35; E35	Gissarskii rozovyi (Bari), Gissarskii rozovyi (Elche)	2	Shared accessions
G05	B15; E16	Cana (Bari), Cana (Elche)	2	Shared accessions
G06	E21; E50	Cranberry (Elche), Koinekasyrskii kislosladkii krasnyi	2	G3 en Zuriaga et al. [13]
G07	E24; E31	Dorosht 5 hahanshahi khoramabad, Entek habi saveh	2	G4 en Zuriaga et al. [13]
G08	E57; E74	Mejhos 6269, Salavatski	2	G5 en Zuriaga et al. [13]
G09	E14; E17	Borde-1 (B113), Casta del reino	2	G6 en Zuriaga et al. [13]
G10	B33; B37	G2, Hicaz	2	
G11	B34; B58	Giardino chiuso dolce, Ottantara	2	
G12	B25; B84	Deve disi, Uzbek	2	
G13	B08; B13	Arakta, Bhagawa	2	
G14	B36; E40	Haku botan, How sweet it is	2	
G15	B51; E42	Myagkosemyannyi rozovyi, Hyrdanar x kirmizy—akbuh	2	
G16	B89; E66	Wonderful P agromillora, Palermo (Elche)	2	
G17	B04; B29; E02	Akko (Bari), Emek, Acco (Elche)	3	
G18	E06; E09; E33; E62	Al-sirin-nar, Apseronski krasnyj, Eve, Nikitski ranni	4	G1 en Zuriaga et al. [13] (+1 accs)
G19	E12; E20; E46; E73	Bala miursal, Crab, Kara bala miursal, Sakerdze	4	G2 en Zuriaga et al. [13]
G20	B02; B21; B48; B69	A dente S. Giorgio, Comune S. Giorgio, Modugno Torrelonga, Reddito dolce	4	
G21	B11; B17; B18; B62; B70; B81	Battista, Capurso acido Surico, Capurso antico Manfredi, Parchitello, S. Giuseppe Moscati, Tardivo acido	6	
G22	B01; B10; B19; B23; B42; B43; B61; B82	A dente Molfetta, Bariblu rotatoria, Capurso dolce Surico, De Marco, Locale Molfetta, Locale Torrelonga, Palese, Tardivo dolce	8	
G23	B22; B24; B49; B60; B67; B68; B88; B90	Cranberry (Bari), Dente di cavallo (Sicilia), Molla nepes (Bari), Palermo (Bari), Purple heart (Bari), Rami turkey, Wonderful, Wonderful (Sicilia),	8	

## Data Availability

All data supporting the findings of this study are fully available within the article and its Supplementary Materials. This includes passport information, morphological descriptors, and genotypic data derived from SSR (Simple Sequence Repeat) markers for the pomegranate accessions conserved in the Elche (Spain) and Bari (Italy) germplasm collections. The complete dataset is provided in the main text and in Supplementary Materials.

## Supplementary Materials

The following supporting information can be downloaded at: https://www.mdpi.com/article/10.3390/plants14213239/s1, Table S1. Data of the 184 analyzed samples, including accession, pomology, ornamental status, origin, type, and germplasm bank; Table S2. Assigned categories for each phenotypic trait, along with a reference accession for each case; Table S3. Shannon-Wiener diversity index calculated per trait and per genebank; Table S4. SSR markers used in this study. Locus code, repeat motif [15], genome position (using Genome assembly ASM765513v2 as reference), melting temperature, fragment length range, number of alleles, PIC, nonbiased expected (Hnb) and observed (Hobs) heterozygosity values are shown; Table S5. SSR marker profiles of the analyzed accessions; Table S6. Bayesian population assignment for each accession at K = 12, indicating the most likely genetic cluster of membership, based on STRUCTURE 2.3.4 software analysis; Figure S1. Spearman correlations among phenotypic traits; Figure S2. Second Factorial Correspondence Analysis (FCAs) showing the names of the accessions analyzed using 16 SSRs. Colors represent the seed hardness. (a) Projection onto Dimensions 1 and 2; (b) Projection onto Dimensions 1 and 3.; Figure S3. Determination of the optimal number of genetic clusters for Bayesian population assignment using STRUCTURE with admixture model. (a) Delta K (ΔK) plot generated with Structure Harvester; (b) mean log-likelihood [L(K)] values and associated variance for each K, also obtained with Structure Harvester; Figure S4. Bayesian population assignment with admixture for K = 12, performed using STRUCTURE software. Each color represents a distinct genetic cluster, and the proportion of each color within an individual’s bar (y-axis) indicates the estimated membership probability in that cluster; Supplementary File S1. Neighbor-Joining tree using Cavalli-Sforza and Edwards’ distance [18] with bootstrap values.
