# Supplementary figures and images for: Pomegranate Germplasm Collections from Elche (Spain) and Bari (Italy): Genetic Resources Characterization for Emerging Mediterranean Challenges
Source: Plants (Basel). 2025 Oct 22;14(21):3239. doi: 10.3390/plants14213239 (PMC12608311; doi:10.3390/plants14213239)

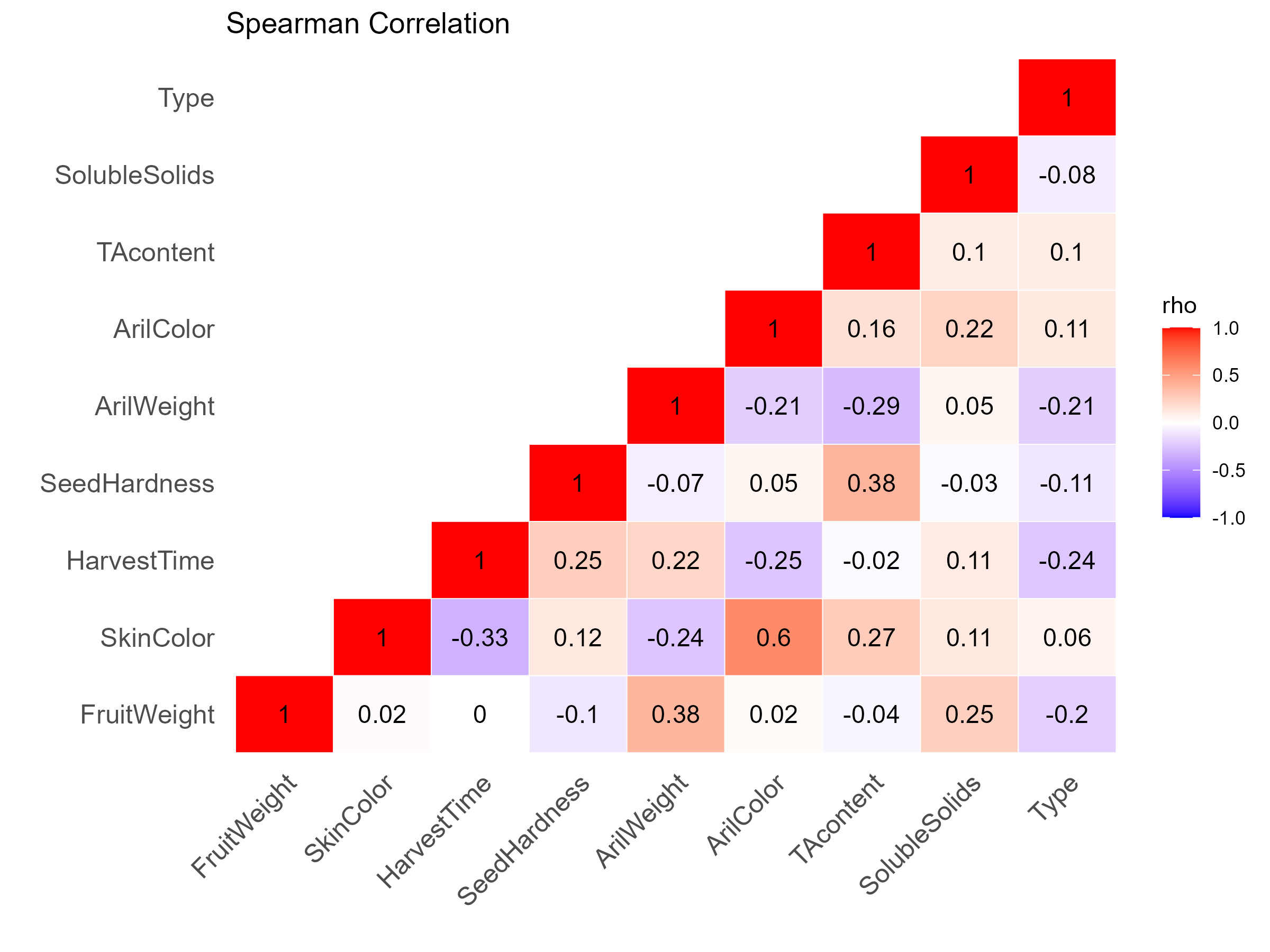

Supplement: Supplementary file 1 [file plants-14-03239-s001.zip › Figure S1.png]

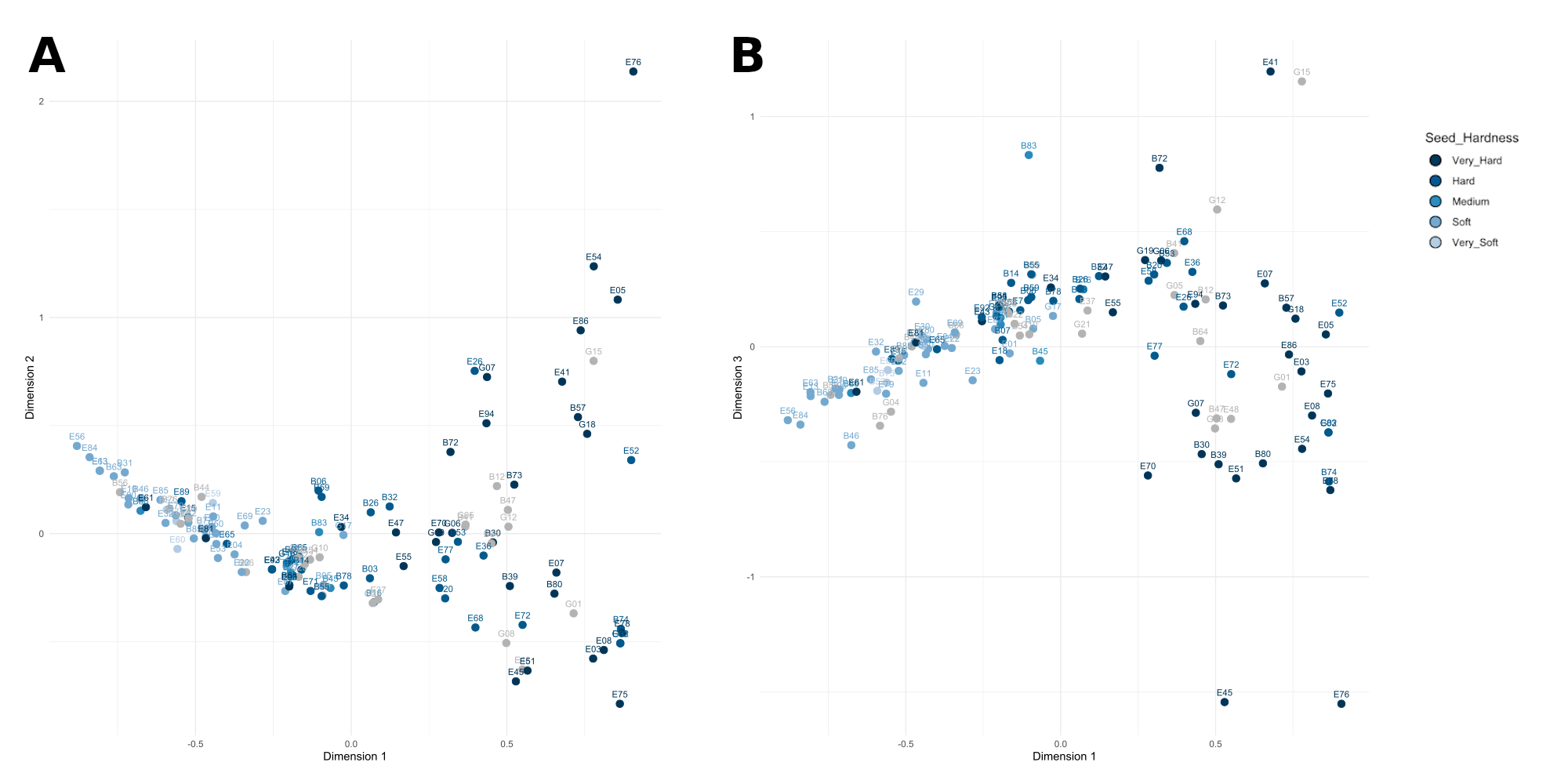

Supplement: Supplementary file 1 [file plants-14-03239-s001.zip › Figure S2.png]

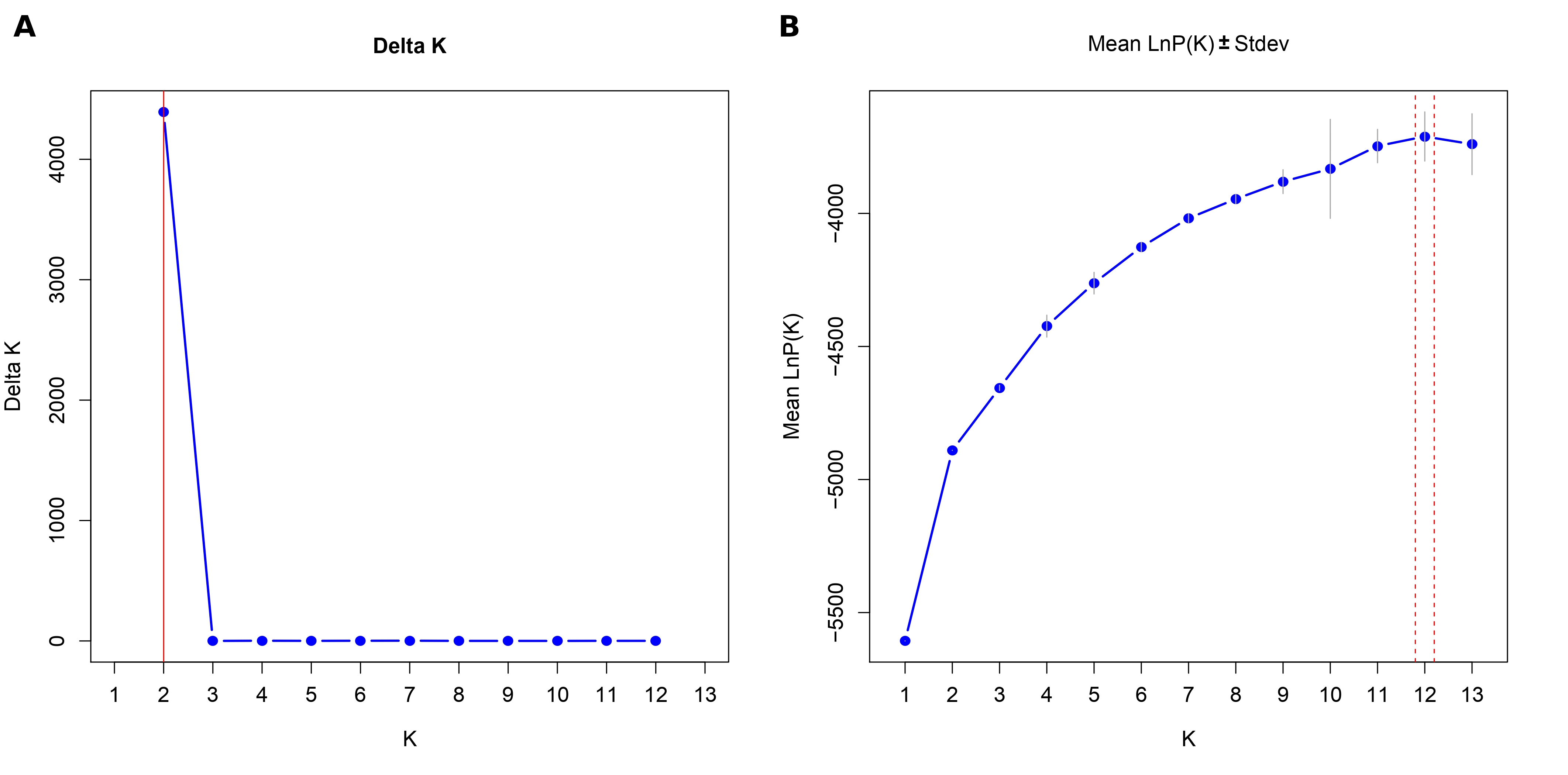

Supplement: Supplementary file 1 [file plants-14-03239-s001.zip › Figure S3.png]

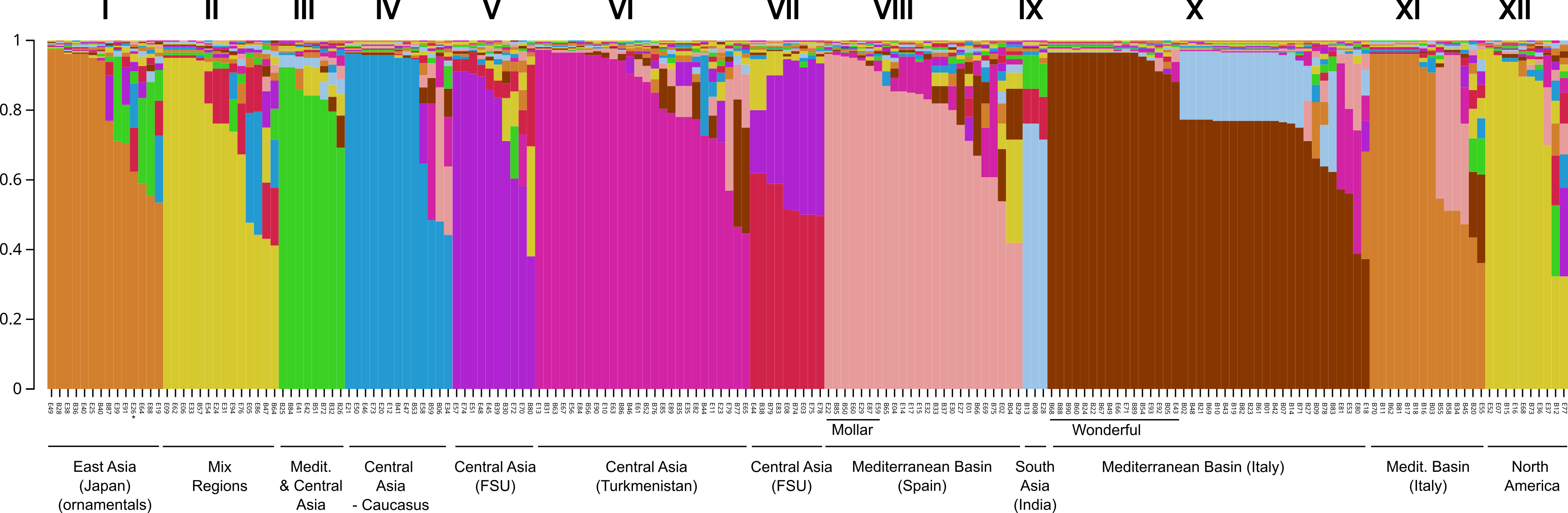

Supplement: Supplementary file 1 [file plants-14-03239-s001.zip › Figure S4.png]
